# Microfluidic Cell Retention Device for Perfusion of Mammalian Suspension Culture

**DOI:** 10.1038/s41598-017-06949-8

**Published:** 2017-07-27

**Authors:** Taehong Kwon, Holly Prentice, Jonas De Oliveira, Nyasha Madziva, Majid Ebrahimi Warkiani, Jean-François P. Hamel, Jongyoon Han

**Affiliations:** 1 Department of Electrical Engineering and Computer Science, Massachusetts Institute of Technology, Cambridge, MA USA; 2 H Prentice Consulting LLC, Carlisle, MA USA; 3 Department of Chemical Engineering, Massachusetts Institute of Technology, Cambridge, MA USA; 4 School of Mechanical and Manufacturing Engineering, University of New South Wales, Sydney, Australia; 5 BioSystems and Micromechanics (BioSyM) IRG, Singapore-MIT Alliance for Research and Technology (SMART) Centre, Singapore, Singapore; 6 Department of Biological Engineering, Massachusetts Institute of Technology, Cambridge, MA USA

## Abstract

Continuous production of biologics, a growing trend in the biopharmaceutical industry, requires a reliable and efficient cell retention device that also maintains cell viability. Current filtration methods, such as tangential flow filtration using hollow-fiber membranes, suffer from membrane fouling, leading to significant reliability and productivity issues such as low cell viability, product retention, and an increased contamination risk associated with filter replacement. We introduce a novel cell retention device based on inertial sorting for perfusion culture of suspended mammalian cells. The device was characterized in terms of cell retention capacity, biocompatibility, scalability, and long-term reliability. This technology was demonstrated using a high concentration (>20 million cells/mL) perfusion culture of an IgG_1_-producing Chinese hamster ovary (CHO) cell line for 18–25 days. The device demonstrated reliable and clog-free cell retention, high IgG_1_ recovery (>99%) and cell viability (>97%). Lab-scale perfusion cultures (350 mL) were used to demonstrate the technology, which can be scaled-out with parallel devices to enable larger scale operation. The new cell retention device is thus ideal for rapid perfusion process development in a biomanufacturing workflow.

## Introduction

In the biopharmaceutical industry, continuous bioprocessing is widely recognized as a next generation biomanufacturing platform for reducing manufacturing cost and improving product quality^1, 2^. Perfusion process is used in bioproduction to achieve high cell concentration (up to 100 million cells/mL) in bioreactors and to enhance volumetric productivity, compared with fed-batch process^3^. In perfusion culture mode, fresh medium is continuously perfused into the bioreactor, and growth-inhibiting metabolites and recombinant products are concurrently removed from the bioreactor using a cell retention device to maintain cells in the bioreactor.

Recent studies have reviewed cell retention devices for the perfusion culture of suspended mammalian cells, including membrane filtration, gravitational settling, centrifugation, and acoustic wave separation^3–8^. The hollow-fiber membrane filter is often used in industry and academia either in the Tangential (cross) Flow Filtration (TFF) or the Alternating Tangential-flow Filtration (ATF) configurations^3–13^. In both systems, a filter module of hollow fibers is externally placed next to a bioreactor, and a pump feeds the cell culture in the bioreactor to the filter module. In TFF, the feed stream flows tangentially on the surface of the hollow-fiber membrane and generates permeate and retentate streams. The permeate stream contains the solute and particles which can move through the pores of the hollow-fiber membrane. The retentate carries the molecules and particles that are too large to pass through the pores.

The hollow-fiber membranes used in the perfusion process, however, are prone to foul due to pore blockage and cake formation by cells and molecules^14^. To reduce membrane fouling and increase the filter lifetime, ATF technology uses a diaphragm to generate rapid and repeated flow cycles between a bioreactor and a membrane module^4, 6, 8–11, 15^. However, ATF remains susceptible to membrane fouling^8, 10, 16, 17^. The fouling becomes more severe as the cell concentration, permeate flow rate, and cultivation time increase, and the viability decreases^10^. Furthermore, high-molecular-weight products generated from cells, such as antibodies and enzymes, may be retained behind the hollow-fiber membrane filter in TFF and ATF^7, 8, 11, 13, 18, 19^ due to membrane fouling and concentration polarization^14, 20^. This potentially diminishes protein recovery and increases the protein residence time in the bioreactor. Also, unwanted smaller dead cells and cell debris produced during cultivation^21^ are retained by the hollow-fiber membrane filter and may release proteolytic enzymes in the bioreactor, possibly affecting productivity^22^ and product quality^23^.

Microfluidic methods for hydrodynamically sorting or separating cells at high-throughput (on the order of a few mL/min per single microchannel) have recently been developed^24^. Inertial microfluidics^25–27^, one of the most successful methods for high-throughput cell sorting^27^, utilizes a combination of hydrodynamic forces dependent on particle size in order to focus and separate particles laterally in a continuous flow within the channel. The control of the motion of particles only requires hydrodynamic forces that are derived from channel structure and particles, without the need for active force fields, such as electric fields or acoustic waves. As such, the inertial microfluidics enables fast, simple and cost-effective cell sorting and separation. Inertial migration in microfluidic channels has previously been applied for the separation of microparticles^28^, isolation of circulating tumor cells^29, 30^, detection of malaria pathogen^31^, and synchronization of cell cycle^32, 33^. Scale-out through parallelization of devices can easily increase the overall flow throughput further (up to 1 L/min)^33–35^. Sensitive cells such as mesenchymal stem cells and leukocytes have been tested in the spiral microchannels demonstrating that this processing does not affect indicators of cell viability, such as membrane permeability and surface proteins^36, 37^. Moreover, it was shown that spiral cell sorting does not induce up-regulation of a shear stress-related gene of the CHO cells^33^.

In this paper, we demonstrate a novel membrane-less cell retention device based on inertial sorting for continuous perfusion culture of suspended mammalian cells. The spiral microchannel with trapezoidal cross-section ^30, 36, 38^ is the basis for a membrane-less cell retention device for perfusion of mammalian cell suspension cultures in this work. We characterized the key performance index of the cell retention device for long-term perfusion culture, in terms of cell retention efficiency, biocompatibility, and scalability. Proof-of-concept perfusion cultures of suspended IgG_1_-producing CHO cells at a concentration of 20–30 million cells/mL were performed over 18–25 days. The comparison of IgG_1_ concentrations between the bioreactor and harvest samples showed that the product recovery efficiency was high (>99%) during the perfusion culture. As an example of a microfluidic cell separation device compatible with large-volume continuous processing systems, the presented work shows a possibility to expand the range and impact of microfluidics applications beyond small-volume diagnostic use.

## Results

### Working principle and design of the microfluidic cell retention device

Neutrally-buoyant particles or cells suspended in a liquid flowing in a straight channel experience a net lift force (F_L_), which is a combination of shear gradient-induced and wall-induced lift forces^25–27^. The curved channels such as spirals generate two counter-rotating flows called Dean vortices perpendicular to the direction of the main flow, inducing an additional drag force (F_D_) on particles or cells^25–27^. Both F_L_ and F_D_ are size-dependent (F_L_ ∝ a^4^ and F_D_ ∝ a, where a is the particle diameter)^27^, and the combination of these forces laterally shifts particles/cells toward a single equilibrium position along the channel.

**Figure 1:**
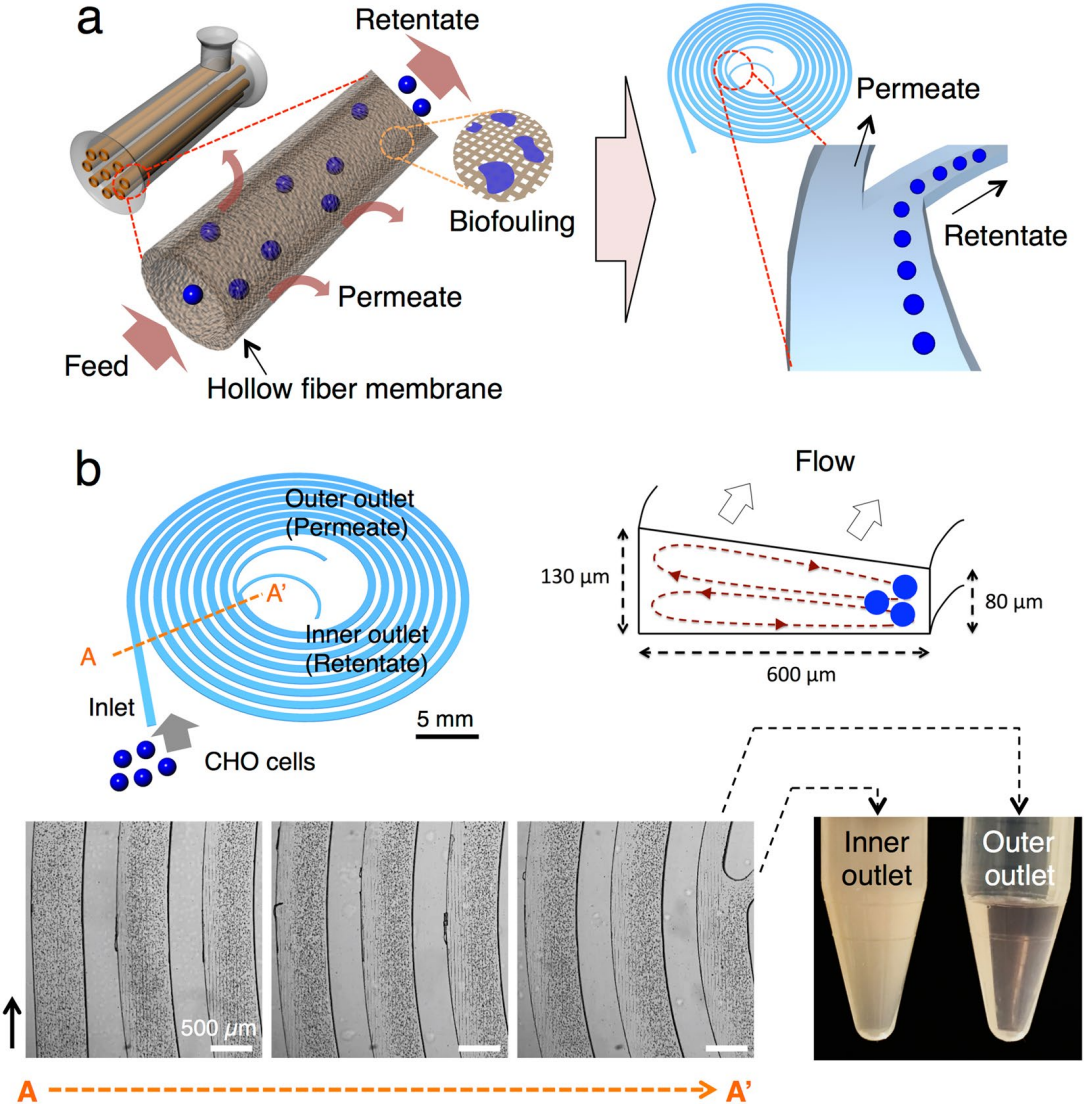
The microfluidic cell retention device. (**a**) Cell retention by hollow fiber membranes and microfluidic cell retention device. (**b**) The schematic of the spiral microfluidic cell retention device. The channel size is 26 mm × 26 mm with 8 loops and its volume is 28 μL. The width is 600 μm, and outer and inner depths are 130 μm and 80 μm, respectively. 10.5 million/mL CHO cells occupied their equilibrium positions near the inner wall and went to the inner outlet. The cell-limited harvest stream flowed into the outer outlet. The two streams from outlets were collected in the tubes. The cell concentration of the harvest stream (outer outlet) had 0.3 million cells/mL with the cell retention efficiency of 97%. Scale bar, 500 μm.

The membrane in ATF and TFF systems retains cells and enables the removal of cell-free by-products (Fig. 1a). Inertial sorting eliminates the need for this membrane, in that cells are sorted away from the junction by only hydrodynamic forces (Fig. 1a).

The spiral microfluidic cell retention device used in this work has one inlet and two outlets (Fig. 1b). The CHO cells of diameter 17.7 μm ± 2.5 μm (mean ± s.d., *n* = 19,346) (Supplementary Fig. S1 and Supplementary Table S1) are initially randomly dispersed in the channel and then begin to occupy their equilibrium position near the inner wall of the channel (Fig. 1b). In this way, the cells are retained through the inner outlet of the channel, and cell-limited harvest is collected through the outer outlet of the channel (Fig. 1b and Supplementary Video S1). When CHO cell culture at the input cell concentration of 10.5 million cells/mL was flowing into the single microfluidic device at the flow rate of 1 mL/min, the collected solution from the outer outlet of the channel was visually clear (0.3 million cells/mL) compared to the solution (12.7 million cells/mL) from the inner outlet (Fig. 1b). This demonstrated the membrane-less cell-solution separation capability of the device.

### Cell retention device characterization

The cell retention device was characterized in terms of cell retention efficiency, biocompatibility, and scalability.

Retention of the cells in a bioreactor through a cell retention device is required to reach and maintain a high productivity. Since the focusing behavior of the CHO cells in the microfluidic cell retention device depends on device dimension, cell culture flow rate, and cell concentration, the optimal operation conditions for high cell retention during perfusion cultures need to be studied. Here we define the cell retention efficiency R as $R( \% )=\frac{{X}_{I}-{X}_{OO}}{{X}_{I}}\times 100$, where *X*_*I*_ is the cell concentration in the bioreactor and *X*_*OO*_ is the cell concentration in the harvest stream. Lower cell concentration in the outer outlet of the device yields higher cell retention efficiency.

**Figure 2:**
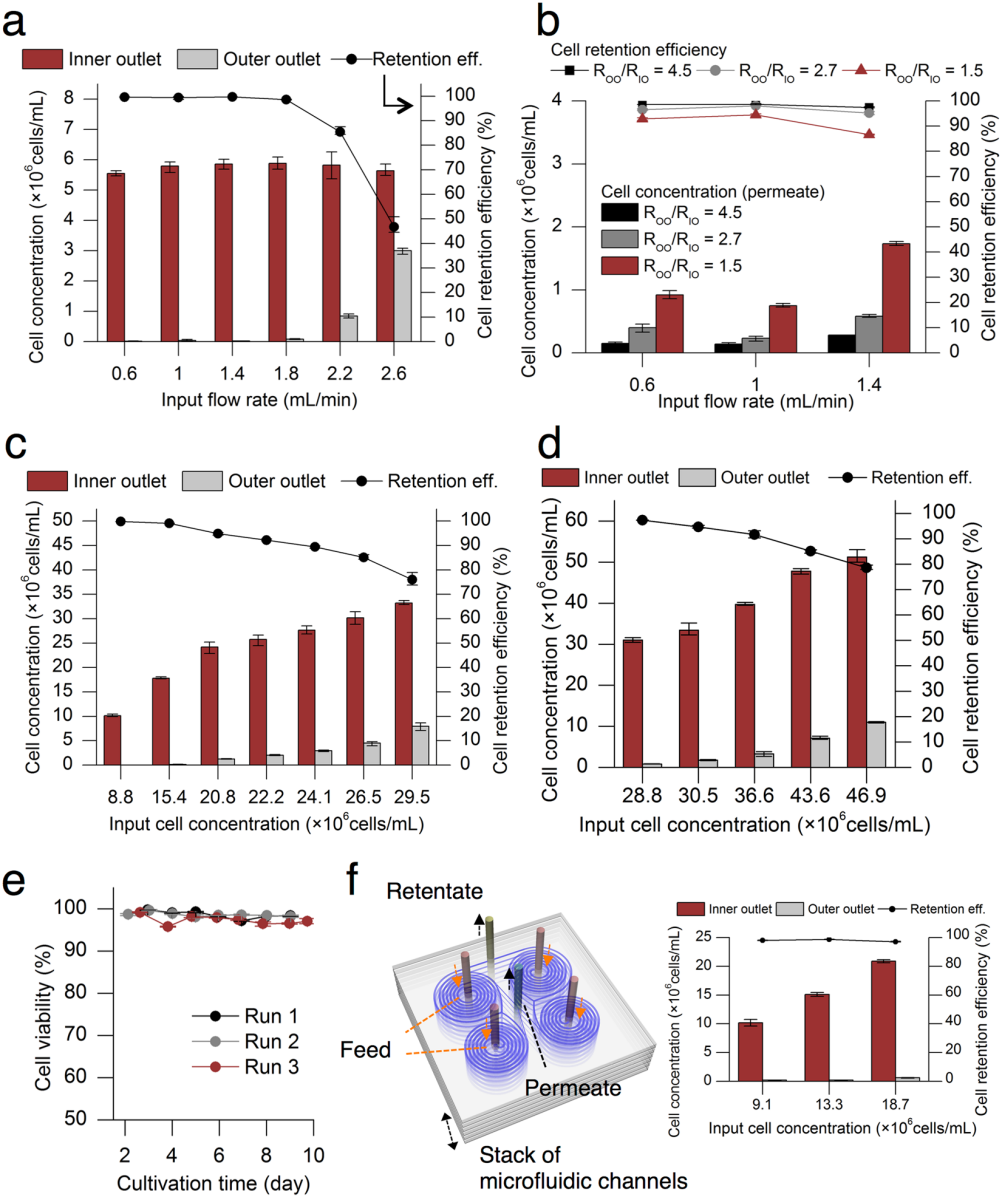
Characterization of the spiral cell retention device in terms of cell retention efficiency, biocompatibility, and scalability. All error bars, data range (*n* = 3) (**a**) The cell retention efficiency can be maintained high at optimal flow rates. The cell culture at the average concentration of 4.8 million cells/mL was flowed into the inlet of the device, and the concentrations of the collected cell culture from the outlets were compared. The input flow rate of 1 mL/min had a cell retention efficiency of 99%. (**b**) Controlling fluid split at the outlets affects retention efficiencies. Decreasing the tubing diameter and increasing the tubing length increased the fluidic resistance of the outer outlet. The cell retention efficiency increased as the ratio of the fluidic resistance of the outer outlet to that of the inner outlet increased. Different R_OO_/R_IO_ values of 4.5 (0.3), 2.7 (0.3), and 1.5 (0.1) (mean (range), *n* = 3 each) were tested. (R_IO_: fluidic resistance of the inner outlet, R_OO_: fluidic resistance of the outer outlet). (**c**) The cell retention efficiency also depends on the input cell concentrations. The device had 91% cell retention efficiency for a cell concentration of 22.2 million cells/mL. (**d**) The cell retention device with a different dimension could improve cell retention capability. The width, inner depth, and outer depth of the channel were 1000 μm, 260 μm, and 80 μm, respectively. The cell retention efficiency was 84% for an input cell concentration of 43.6 million cells/mL. The input flow rate and fluidic resistance ratio were 4 mL/min and 1:0.1 (outer outlet:inner outlet), respectively. (**e**) The CHO cells (4.2 million cells/mL on average) were continuously processed by the single cell retention device with the average retention efficiency of 98% for >145 hours at the flow rate of 1 mL/min. The noticeable decrease in viability was not observed during three different runs. (**f**) Schematic diagram of scaled-out microfluidic cell retention technology and the cell retention performance of the five-layer stacked device. The four spiral channels are combined in one single layer, and the layers can be stacked to increase a perfusion rate.

Given a fixed channel dimension (600 μm width, 80 μm inner depth, and 130 μm outer depth), the cell retention efficiency depends on input flow rate, fluidic resistance ratio at the outlets, and input cell concentrations (Fig. 2a–c). The CHO cells were focused near the inner wall of the channel at the flow rate of <1.5 mL/min, and this resulted in high cell retention efficiencies of 99.6% ± 0.3% (mean ± s.d., *n* = 9) at the input concentration of 4.8 million cells/mL (Fig. 2a). As the flow rate increased, the smaller CHO cells (<10 μm) started to shift their equilibrium positions near the outer wall of the channel, leading to loss of these smaller cells into the outer outlet of the device and reduced cell retention efficiencies of 85% (3%) and 46% (6%) (mean (range), *n* = 3) at the flow rates of 2.2 and 2.6 mL/min, respectively (Fig. 2a).

The fluidic resistance ratio at the outlets also affected the cell retention efficiency by modulating the streamline boundary between two flows for the inner outlet and outer outlet at the channel bifurcation (Fig. 2b and Supplementary Fig. S2). The higher fluidic resistance for the outer outlet than that for the inner outlet moved the streamline boundary closer to the outer outlet so that the flow for the inner outlet contained more focused cells than that for the outer outlet in the main channel (Supplementary Fig. S2), increasing the cell retention efficiency.

The cell retention efficiency in this microfluidic cell retention device also depends on the input cell concentration. Increased input cell concentrations result in increased cell-to-cell interaction because the cells compete with each other for the same equilibrium position along the channel. This led to broadening of the focused band and decreased efficiency of inertial focusing (Fig. 2c). The microfluidic device had 99.2% (0.1%) (mean (range), *n* = 3) cell retention efficiency for a cell concentration of 15.4 million cells/mL and 92.2% (0.8%) (mean (range), *n* = 3) cell retention efficiency for a cell concentration of 22.2 million cells/mL (Fig. 2c). However, the cell retention efficiency drops below 83% for the concentrations greater than 26.5 million cells/mL due to broadened focusing bands, resulting in cell loss to the outer outlet of the device. Still, the achieved level of cell retention was sufficient to enable long-term perfusion culture with >20 million cells/mL, with continuous cell bleeding (see below). The cell retention efficiency for the higher cell concentration such as 30–40 million cells/mL can be improved with the modified channel dimension and flow rates (Fig. 2d).

The cell retention devices need to be in a continuous operation, during long-term perfusion culture, without negatively affecting the cell growth and viability to maintain high cell concentrations in the bioreactor. The CHO cells at 4.2 million cells/mL (1.2 million cells/mL) (mean (range), *n* = 3) were continuously flowed through the cell retention device for more than 145 h, and processing of the cells through the retention device for a long-term did not impact cell viability (Fig. 2e), while maintaining a cell retention efficiency of 98% (1.6%) (mean (range), *n* = 3). Moreover, the perfusion culture using the retention device maintained the same average specific growth rate (0.03 h^−1^) during exponential growth phase as that from batch cultures (See below for the perfusion cultures and Supplementary Table S2).

The throughput of the new cell retention technology needs to be high enough to process the large volumes (several thousand liters) from large-scale bioreactor tanks. The spiral inertial microchannels can be easily scaled-out using multiple devices in parallel (Fig. 2f). The five-layer stacked device showed cell retention performance similar to that of a single-layer device (Fig. 2c and f). With the fluidic resistance ratio (inner outlet:outer outlet = 1:4) adjusted for the cell retention and the single input flow rate of 1 mL/min, the current single device can process the perfusion rate of 288 mL/day, and the four devices in parallel can handle the perfusion rate of 1.15 L/day. With the modified design, the perfusion rate of a wider and deeper single chip can at least quadruple in that the input flow rates for cell sorting can be increased by four fold (Fig. 2d). As a demonstration of scalability of the cell retention technology, four spiral channels were combined in a single layer to perfuse 700 mL/day during perfusion cultures (see below).

### Perfusion culture using the spiral cell retention device

**Figure 3:**
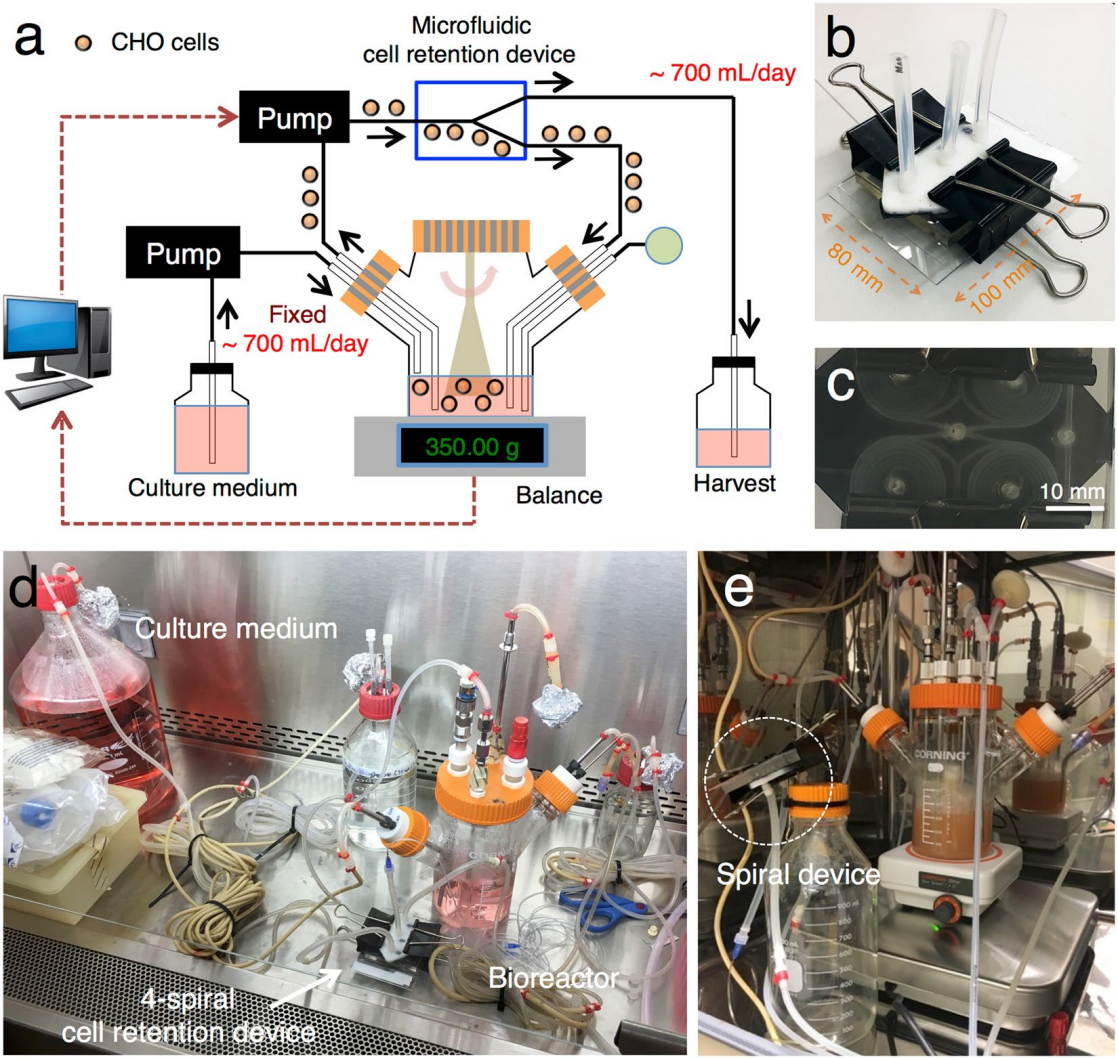
The perfusion culture setup using the spiral microfluidic cell retention device. (**a**) The cell culture was continuously processed through the device by a peristaltic pump. The majority of the cells were recycled back to the bioreactor while the harvest was collected in a separate container. The cell culture medium was supplied to the bioreactor using another peristaltic pump. The working volume of the bioreactor was controlled automatically based on bioreactor weight using customized software. The pH and dissolved oxygen levels were monitored and controlled to meet established set points. (**b**) The microfluidic cell retention device and the fluidic adaptor. (**c**) The bottom view of the cell retention device. The four spiral channels were combined in parallel. Scale bar, 10 mm. (**d**) The pre-sterilized cell retention device, tubings, and bottles were connected to each other in a sterilized manner in the biosafety cabinet. (**e**) The setup was transferred into the standard CO_2_ incubator, and the perfusion culture began after four days.

In this work three sequential perfusion cultures of suspended CHO cells using the spiral cell retention device were carried out, as described in the Methods (Fig. 3). The IgG_1_-producing CHO cells were grown in a customized bioreactor with the working volume of 350 mL. The cells were continuously flowed through the cell retention device using a peristaltic pump, and most of the cells (98.4% ± 1.1% (mean ± s.d., *n* = 15) in terms of total cell number) were recycled back to the bioreactor. The harvest solution containing IgG_1_ was collected from the outer outlet of the device. At the same time, fresh culture medium was supplied to the bioreactor to maintain a constant working volume.

**Figure 4:**
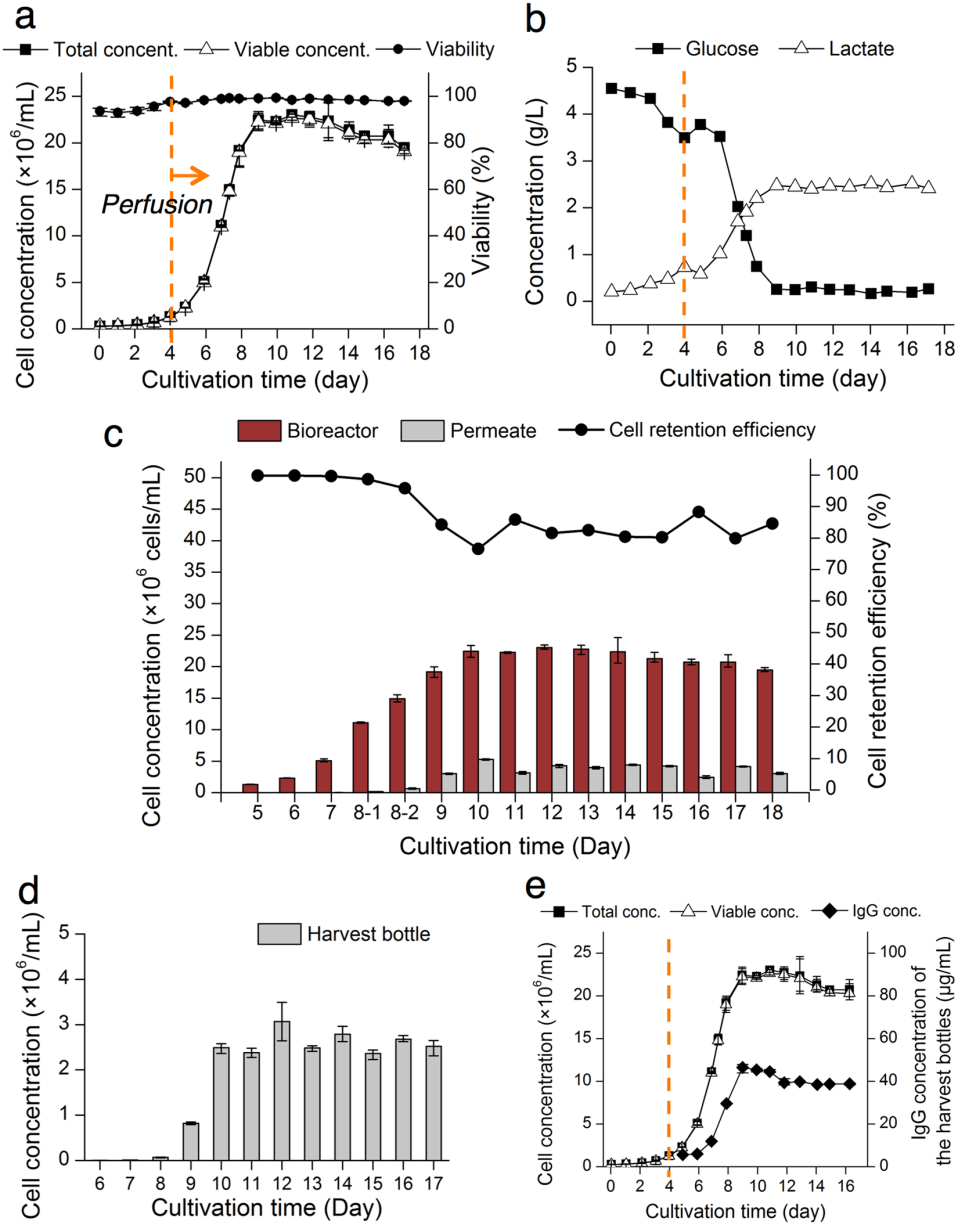
The perfusion culture results. All error bars, data range (*n* = 3, technical replicates). (**a**) The bioreactor culture of the CHO cells was performed for 18 days. The perfusion began after the 4-day batch culture. The cells continued to proliferate until the cell concentration peaked at 22 million cells/mL. The leveled-off concentration was 20 to 23 million cells/mL. The viability was maintained >98% after the cells became saturated. (**b**) The glucose and lactate levels were stabilized after the perfusion began. The stabilized glucose and lactate concentrations were 0.24 g/L and 2.46 g/L, respectively. No glucose spiking was done in this perfusion culture. The data points are average values from technical replicates (*n* = 2). (**c**) The cell concentrations of the bioreactor and the device outer outlet (permeate) were compared daily to measure cell retention efficiencies of the device. The cell retention device maintained the average cell retention efficiency of 82% for 20 to 23 million cells/mL throughout the perfusion culture. (**d**) The cell concentration (2.6 million cells/mL on average since Day 10) of the daily collected harvest bottles confirmed the continuous stable cell retention of the device. (**e**) The production of IgG_1_ was demonstrated in the perfusion culture.

**Figure 5:**
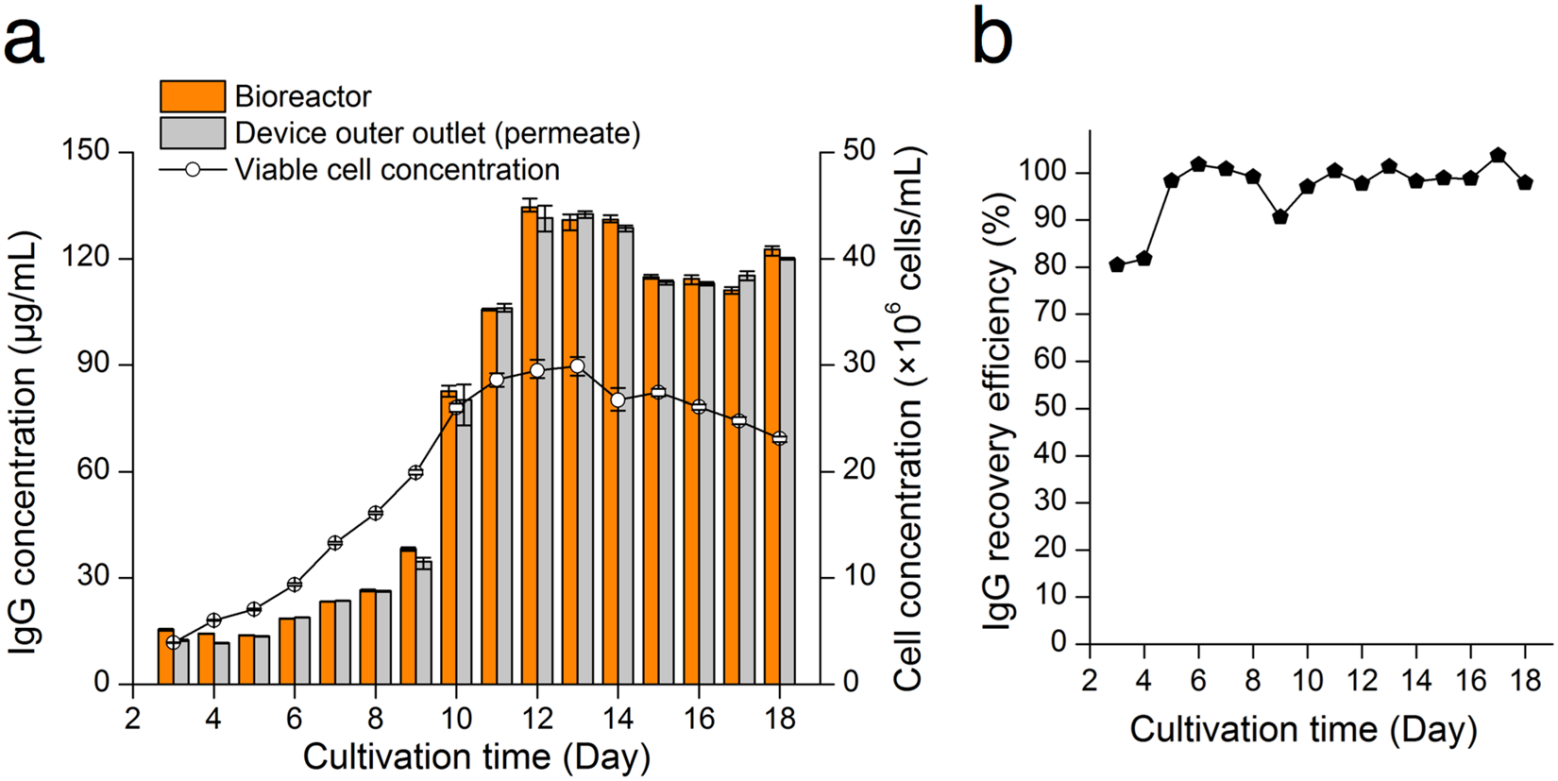
Antibody recovery by the microfluidic cell retention device. (**a**) Antibody concentrations from the bioreactor and outer outlet (the permeate stream) of the device were compared daily. Error bars, data range (*n* = 3, technical replicates). (**b**) The recovery efficiency close to 100% demonstrates that the microfluidic cell retention device does not suffer from the low product recovery, compared with the membrane-based filtration technologies.

The three separate bioreactor cultures were run for 18 to 25 days (Figs 3–5 and Supplementary Figs S3–S5). In the first bioreactor culture, the CHO cells were grown in a batch mode (no perfusion) for the first four days, followed by the initiation of the perfusion culture at 2 VVD (vessel volume per day). The cell concentration reached 22.7 million cells/mL by Day 10 and leveled off afterward (Fig. 4a), exceeding that from the batch cultures using the same cell line by four times (Supplementary Table S2). The peak cell concentration obtained was likely a result of limited nutrients and oxygenation (Fig. 4b and Supplementary Fig. S3), as well as increased “bleeding” of cells (reduced cell retention efficiency) at high cell concentration. Still, cell viability was maintained >97% after the perfusion began, and it was 99% ± 1% (mean ± s.d., *n* = 9) after the cell concentration peaked on Day 10 (Fig. 4a), confirming the long-term biocompatibility of the microfluidic cell retention device. The culture metabolic parameters such as glucose, lactate, glutamine, glutamate, and ammonium leveled off during the perfusion phase of the culture (Fig. 4b and Supplementary Fig. S3). The glucose concentration was stably maintained at 0.24 g/L after Day 10, creating a glucose-limited environment that was still able to support the high cell concentration in the bioreactor.

We compared the cell concentration in the bioreactor with that of the harvest stream (supernatant coming from the device outer outlet) on a daily basis (Fig. 4c). The cell retention efficiency was 99% ± 2% (mean ± s.d., *n* = 5) for cell concentrations <15 million cells/mL in the bioreactor. As the culture reached its peak concentration of 20 to 23 million cells/mL, the average cell retention efficiency was 82% ± 3% (mean ± s.d., *n* = 10). The low cell number lost to the harvest bottle demonstrates that the cell retention through the spiral device was maintained throughout the culture (Fig. 4d). During this perfusion culture, the spiral cell retention device did not show any noticeable cell accumulation (Supplementary Fig. S4).

IgG_1_ production efficiency of the perfusion culture was also evaluated. The CHO cells in the bioreactor continuously produced recombinant IgG_1_, and the IgG_1_ concentration in the bioreactor increased with cell concentration (Fig. 4e) and peaked at approximately 40 µg/mL after Day 9. The total harvest volume of 8.16 L during perfusion was collected, with a total IgG_1_ yield of 263 mg. The IgG_1_ production rate was 26.8 mg/day from Day 9.

The culture data demonstrated the feasibility for the use of the microfluidic cell retention device in mammalian perfusion culture. Further studies are required to demonstrate the use of the device for large-scale perfusion.

### Product recovery with the cell retention device

Since the spiral cell retention device does not have any micro- or nanoporous membrane, and the smallest channel dimension is in the order of ~100 µm, the recombinant product generated from the cells can be directly collected without any retention or loss during perfusion cultures. To demonstrate high product recovery with the microfluidic device, another perfusion culture using the spiral cell retention device was performed for 18 days (Fig. 5a and Supplementary Fig. S5), and the IgG_1_ concentrations in the bioreactor and outer outlet of the device were measured. Both concentrations were similar during the perfusion culture (Fig. 5a). Antibody recovery efficiency was calculated as $AR( \% )=\frac{{X}_{OO}}{{X}_{I}}\times 100$, where *X*_*I*_ is the IgG_1_ concentration in the bioreactor (feed), and *X*_*OO*_ is the IgG_1_ concentration in the harvest stream (permeate). The IgG_1_ recovery efficiency was only 80% for the first two days, possibly due to adsorption of IgG_1_ to the tubing and fluidic adaptors, and then increased close to 100% thereafter (Fig. 5b).

## Discussion

Other membrane-less cell retention technologies such as acoustic wave-based cell aggregation, centrifugation, and inclined sedimentation are available^3, 4, 6, 8^. Acoustic cell retention requires additional active components, such as high frequency (~2 MHz) ultrasonic transducers^7^. Centrifuges can create high shear stress on cells and have high operation and maintenance costs^4^, thus is not an ideal choice for continuous biomanufacturing. Inclined sedimentation has limitations of long residence time of cells in the settler and low cell retention efficiency for high cell concentrations^8^. In contrast, our inertial sorting based microfluidic cell retention process is a simple, passive operation (no active field) with minimal maintenance needed (no filter/device replacement), and benign to cells with high cell viability demonstrated. Supplementary Table S3 shows a comparison among the spiral microfluidics and other cell retention technologies in terms of cell retention efficiencies, scalability, product recovery, dead cell removal, and capital and operation costs.

Using the spiral cell retention device, we demonstrated perfusion cultures of the CHO cells with the concentration of 20 to 30 million cells/mL using a commercial media formulation. With additional device optimization in terms of different device dimensions (*e.g*., large slant angle of the trapezoidal cross-section for increasing cell trapping capability^38^ and optimized bifurcated outlets), enabled by model-based engineering, the cell retention capability of the device can be further enhanced. Moreover, cascading multiple devices increases cell retention efficiency at high cell concentrations^34, 39, 40^. As the viscoelasticity of the cell culture rises with increasing cell concentration^41^, viscoelastic inertial microfluidics^25–27, 42^ will be useful to understand the focusing behavior of the high-concentration CHO cells and enhance the cell retention capability of the spiral cell retention devices. Furthermore, combination of other passive microfluidic cell separation technologies, such as vortex-assisted cell separation^43–45^, is worthwhile for future work. The aforementioned approaches may not be sufficient to process high cell concentration of >100 million cells/mL. However, whether the cell concentration during bioreactor cultivation should be increased to >100 million cells/mL remains unclear. This high cell concentration could result in issues, such as decreased mixing efficiency due to high medium viscosity, limited oxygenation, and high carbon dioxide accumulation^46, 47^, thus requiring sophisticated aeration and agitation strategies for reliable long-term biomanufacturing. Therefore, we believe that microfluidic cell retention below 100 million cells/mL is still meaningful.

For large-scale perfusion culture (>100 L/day perfusion rate), the scale-out of the microfluidic cell retention devices using parallelization is required. Assuming a large spiral channel (1000 μm width, 260 μm inner depth, and 80 μm outer depth of the channel) with 4 mL/min input flow rate and 0.4 mL/min harvest flow rate at high cell concentrations (>40 million cells/mL), 1.7 channel is required for 1 L/day perfusion rate. For example, approximately 600 channels are needed to process 1,000 L/day perfusion rate. The overall dimensions of this stacked device are 100 mm × 80 mm × 300 mm, assuming a single layer contains four spiral channels and is 2 mm thick. Enlarging channel dimensions while retaining cells under inertial microfluidics remains possible and can further reduce the number of spiral channels for decreased footprint. The stacked devices can also theoretically achieve uniform flow distribution because the common inlet and outlet paths (4 mm in diameter each) have significantly lower fluidic resistance than the dimensions of the spiral channel. Flow distribution in a 10-layered device was simulated, and uniform flow rates across each layer was demonstrated (Supplementary Fig. S6). The large number of the spiral channels (*e.g*., 600 channels for 1,000 L/day perfusion rate) is expected to contribute to stable cell retention during long-term cultures by minimizing the risk of the channel failure possibly due to large cell–cell debris aggregates. If one channel loses all cells to the harvest stream, the cell retention efficiency of the stacked device decreases by 1% but the fluid split ratio remains nearly the same, assuming the intact stacked device has 90% cell retention and 9:1 (retentate:harvest) fluid split ratio.

At the high cell concentration (>20 million cells/mL) during perfusion using the conventional membrane-based filtration devices, 5 to 10% daily cell bleeds are commonly used to offset growth and maintain target cell densities at high cell viability, thereby achieving a steady state culture. The microfluidic cell retention device can also perform continuous cell bleeding at high cell density with no need for manual intervention because a fraction of the cells can be removed from the bioreactor through the harvest flow (Fig. 4c,d and Supplementary Fig. S5). Moreover, the cell bleeding efficiency, as well as the target cell concentration, can be controlled via changing various parameters, such as input flow rate into the device, channel dimensions, and outlet fluidic resistance ratio (Fig. 2a–d). In addition, our spiral cell retention device based on inertial sorting can be configured to separate cells of different sizes with the specific input flow rates^33, 38^. Thus, the smaller non-viable cells with the cell debris could be continuously removed in the outflow, which provides a favorable culture environment for cell growth and possible benefits to the culture (*e.g*., enhanced product quality^23^ and productivity^22^).

In the current study, we did not use a highly productive cell line or optimize the culture conditions to maximize mAb productivity. The mAb productivity of the CHO cells is known to be affected by many parameters, such as CO_2_ concentration in the bioreactor, culture temperature, and cell lines. Using our system, perfusion cultures with different culture parameters and cell lines could be performed to study the mAb productivity of the current CHO cell lines, at the lab scale (<0.5 L) perfusion culture volume. To the best of our knowledge, this work is the first demonstration of a benchtop scale (350 mL), high cell density, long-term perfusion culture using a microfluidic device, which can be applied to pharmaceutical process development and manufacturing.

Adjusting the fluidic resistance for the harvest stream using pinch or multiple-way valves enables the modulation of perfusion rates during cultivation. Kim and *et al*. demonstrated a long-term modulation of fluidic resistance using automated pinching/releasing mechanism^48^. In addition, multiple-way valves can be connected to the harvest stream so that users can choose different fluidic paths with varied fluidic resistances. If the modulation of fluidic resistance ratio at the outlets significantly affects cell retention efficiency, then users can increase the number of spiral chips to process the target perfusion rates while maintaining the fluidic resistance ratio for each spiral channel.

Also, the spiral cell retention device tested here was made of polydimethylsiloxane, a low-cost platinum-cured silicone commonly used in pharmaceutical and bioprocessing industries^49, 50^. The dimensions of the microfluidic channels (~100 µm) are well within the limit of standard plastics manufacturing processes (*i.e*., injection molding using polystyrene), with the possibility of significantly lowering the unit cost.

As the membrane-less microfiltration based on inertial sorting is applicable to other microorganisms such as yeast and bacteria^33^, interesting applications such as production of biofuels and biochemicals using smaller microorganisms^51^ could be also contemplated.

Lastly, it is expected that membrane-less microfiltration based on inertial microfluidics will find many other industrial applications by separating large scale suspension efficiently and at lower cost than conventional membranes^35^.

## Methods

### Fabrication of cell retention devices

Microfluidic channels were fabricated using standard soft lithography. The molds for microfluidic channels were designed using a 3D modeling tool (Rhinoceros, McNeel North America, USA). The single spiral channel (600 μm width, 80 μm inner depth, and 130 μm outer depth) possessed eight loops and an outlet width of 300 μm. The four spiral channels in parallel possessed six loops and an enlarged width (520 μm) for the inner outlet. The aluminum channel molds were fabricated using micromachining (Whits Technologies, Singapore). Polydimethylsiloxane (PDMS) elastomer (Sylgard^®^ 184, Dow Corning, USA) was prepared and the solution was poured into the aluminum molds and cured at 150 °C for 15 min on a hotplate. The solidified patterned PDMS slab was removed from the mold and punched with a 4 mm puncher to make inlets and outlets. The slab was bonded to a flat glass slide (260230, Ted Pella, USA) or thin PDMS layer (<500 um) using oxygen plasma treatment (Harrick Plasma Cleaner, Harrick Plasma, USA). The assembled PDMS microchannel was cured at 95 °C overnight on a hotplate. Silicone tubings (Masterflex, Cole-Parmer, USA) for fluid transfer were inserted into inlets and outlets of the microchannel the next day. Finally, the glass or acrylic slide with drilled holes was placed on top of the microchannel, and they were clamped with binder clips for long-term robust operation.

### Cell batch culture

CHO-DG44 cells producing human IgG_1_ against CD40 ligand were gifted from Biogen Idec, MA, USA. Commercial culture medium (12681011, CD OptiCHO^TM^, Thermo Fisher Scientific, USA) was used with 50 µM of L-methionine sulfoximine (M5379, Sigma-Aldrich, USA). Suspension cultures were performed in an autoclaved spinner flask (4500-500, PYREX ProCulture Spinner Flask, Corning, USA) on a magnetic stirrer. Cell concentrations, viabilities, live cell diameters, pH values, gases, electrolytes, nutrients, and metabolites were measured by the automated analyzer (BioProfile FLEX and CDV Analyzers, Nova Biomedical, USA). The cells from a sample cell culture are spread in a single layer in a chamber, and live cells are marked by trypan blue dye exclusion. For each measurement, 40 images are obtained from 40 different fields of view. Their diameter is determined by measuring a pixel distance, and the cell concentration is measured by counting cells in images. For example, 6,000 and 12,000 CHO cells are captured to obtain total cell concentrations of 5 and 10 million cells/mL, respectively.

### Device characterization

Several tubes (Falcon Centrifuge Tube, Corning, USA) of the cells were combined after centrifugation to create high cell concentrations (>8 million cells/mL). The cell retention device was mounted on an inverted microscope (IX51, Olympus, USA), and cells were loaded into a syringe (BD Luer-Lok^TM^ tip syringe, Becton, Dickinson and Company, USA). Subsequently, a syringe pump delivered the cell solution to the device. The focusing behavior of CHO cells in the microfluidic cell retention device was captured by a high-speed camera (Phantom V9.1, Vision Research, USA). To measure the cell retention efficiency, three sets of both inner and outlet samples in tubes were obtained sequentially per experimental condition. Afterward, the cells collected from both outlets were analyzed by the automated equipment (Bioprofile CDV Analyzer, Nova Biomedical, USA) to obtain cell concentration and viability. The maximum deviation from mean concentration was 4.8% ± 3.7% (mean ± s.d., *n* = 32) for cell concentration of >1 million cells/mL. Input cell concentrations were estimated using volume ratios and average cell concentrations of outlet samples. The fluidic resistance ratio at the outlets was modified by attaching a small-diameter (inner diameter, 510 µm) tubing (EW-06420-02, Cole-Parmer, USA) and adjusting its length. A resistance ratio was calculated as the ratio of the weight of solutions collected from each outlet. For long-term viability measurement, the cell culture in a spinner flask with a working volume of 250 mL was continuously recycled through the cell retention device at a flow rate of 1 mL/min. The cell culture in the flask was perfused with a fresh culture medium at 0.2 mL/min, and the working volume was maintained without pH and dissolved oxygen (DO) controls. The daily cell culture sample was aliquoted into three vials, and concentration and viability were measured by the automated equipment (Bioprofile CDV Analyzer, Nova Biomedical, USA).

### Perfusion culture

A spinner flask was customized for DO and pH controls. The flask with pH/DO probes and tubings was sterilized using autoclave. The setup was connected in a biosafety cabinet to the presterilized glass bottles (culture medium, base solution, cell inoculation, and harvest collection). The microfluidic retention device was sterilized by flushing the channel with 10% bleach and 70% ethanol for at least 10 min using a peristaltic pump (07522-20, Masterflex, USA). Subsequently, the device was connected to the sidearm ports of the flask and harvest bottle. Afterward, 400 mL of the prewarmed sterilized culture medium was transferred into the flask. Finally, the whole setup, except for the base solution and harvest bottles, was moved into standard CO_2_ incubators to maintain the stable temperature at 37 °C. The flask was placed on a magnetic stirrer (440811, Corning, USA), and the stirring revolution per minute (RPM) was 45 throughout the culture. Bioreactor controllers (BioFlo/CelliGen 115 systems, New Brunswick Scientific, USA; BIOSTAT A bioreactor controller, Sartorious, USA) were used to control pH (7.0) and DO (40%, with respect to air saturation). The CHO cells were inoculated by a peristaltic pump from the inoculation bottle to the spinner flask. The initial cell concentration was 0.3–0.4 million cells/mL, and the cells were grown in a batch mode prior to perfusion. During perfusion of culture, fresh culture medium and cell broth were fed in the spinner flask and the microfluidic retention device, respectively, by using two peristaltic pumps (07522-20, Masterflex, USA; 120U/DV, Watson Marlow, USA). The working volume of the culture was maintained at approximately 350 mL throughout the perfusion of culture using a customized control system. The harvest bottle was replaced daily during perfusion. Cell concentration was measured from technical replicates (three aliquots). The maximum deviation from mean concentration was 3.8% ± 2.9% (mean ± s.d., *n* = 64) for cell concentration of >1 million cells/mL.

### Antibody concentration measurement

The supernatant antibody concentrations were determined by performing a high-performance liquid chromatographic assay using an affinity chromatography column (2-1001-00, Applied Biosystems, USA) on a liquid chromatography system (1100 Series, Agilent, USA). An equilibration/wash buffer of pH 7 contained 50 mM phosphate (Sigma-Aldrich, USA) and 150 mM sodium chloride (Sigma-Aldrich, USA). An elution buffer of pH 2.5 contained 100 mM phosphate and 0.4% (v/v) of phosphoric acid (85 wt%, Sigma-Aldrich, USA). The purified IgG_1_, Kappa from human myeloma plasma (I5154, Sigma-Aldrich, USA) was used to generate a reference standard curve.

### Data availability

All the raw data were stored in a laboratory server and concurrently backed up to remote locations. The data will be made available in response to any reasonable requests, after the publication and completion of necessary intellectual property-related steps.

## Electronic supplementary material

Supplementary Video S1

Supplementary Information
