# Supplementary material for: Microfluidic Cell Retention Device for Perfusion of Mammalian Suspension Culture
Source: Sci Rep. 2017 Jul 27;7:6703. doi: 10.1038/s41598-017-06949-8 (PMC5532224; doi:10.1038/s41598-017-06949-8)
Supplement: Supplementary file 2 — Supplementary Information [file 41598_2017_6949_MOESM2_ESM.pdf]

Supplementary Information

Microfluidic Cell Retention Device for Perfusion of Mammalian Suspension Culture

Taehong Kwon[1], Holly Prentice[2], Jonas De Oliveira[3], Nyasha Madziva[3], Majid Ebrahimi Warkiani[4], Jean-François. P. Hamel[3**], and Jongyoon Han[1, 5, 6*]

[1]*Department of Electrical Engineering and Computer Science, Massachusetts Institute of Technology, USA*

[2]*H Prentice Consulting LLC, USA*

[3]*Department of Chemical Engineering, Massachusetts Institute of Technology, USA*

[4]*School of Mechanical and Manufacturing Engineering, University of New South Wales, Australia*

[5]*BioSystems and Micromechanics (BioSyM) IRG, Singapore-MIT Alliance for Research and Technology (SMART) Centre, Singapore*

[6]*Department of Biological Engineering, Massachusetts Institute of Technology, USA*

[*]*Correspondence should be addressed to Jongyoon Han (jyhan@mit.edu)*
Phone : +1-617-253-2290, Fax : +1-617-258-5846

[**]*Co-correspondence should be addressed to Jean-François. P. Hamel (jhamel@mit.edu)*
Phone : +1-617-258-6665, Fax : +1-253-9894

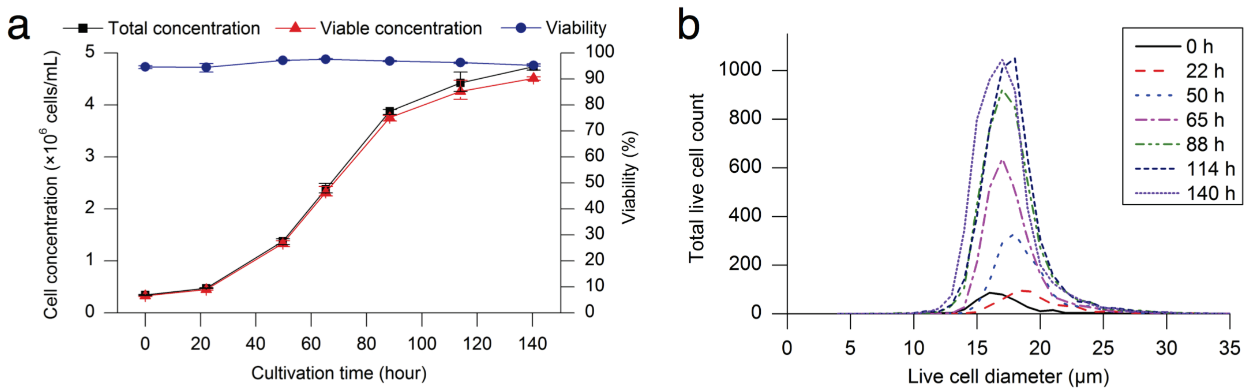

Supplementary Figure S1 Batch culture and distribution of diameters of live CHO cells. **(a)** CHO cells were cultivated in a spinner flask on batch mode for 140 h. Concentration and viability were analyzed daily using automated equipment (Bioprofile CDV Analyzer, Nova Biomedical, USA). The total cell concentration reached approximately 4.7 million cells/mL, and the viability was 95% at 140 h. Error bars, data range (*n* = 3, technical replicates). **(b)** Diameters of live CHO cells were measured using the same equipment. The cells had an average diameter of 17.8 μm during growth phase (*e.g.*, 65 h).

Cultivation time (h)	Number of live cell cells analyzed	Average live cell diameter (μm)	Standard deviation of live cell diameters (μm)
0	380	17.2	2.7
22	502	19.7	3.1
50	1,458	18.7	2.5
65	2,617	17.8	2.4
88	4,370	17.7	2.5
114	4,811	17.8	2.4
140	5,208	17.1	2.4
**Total**	**19,346**	**17.7**	**2.5**

Supplementary Table S1 Summary of data in **Supplementary Figure S1b**.

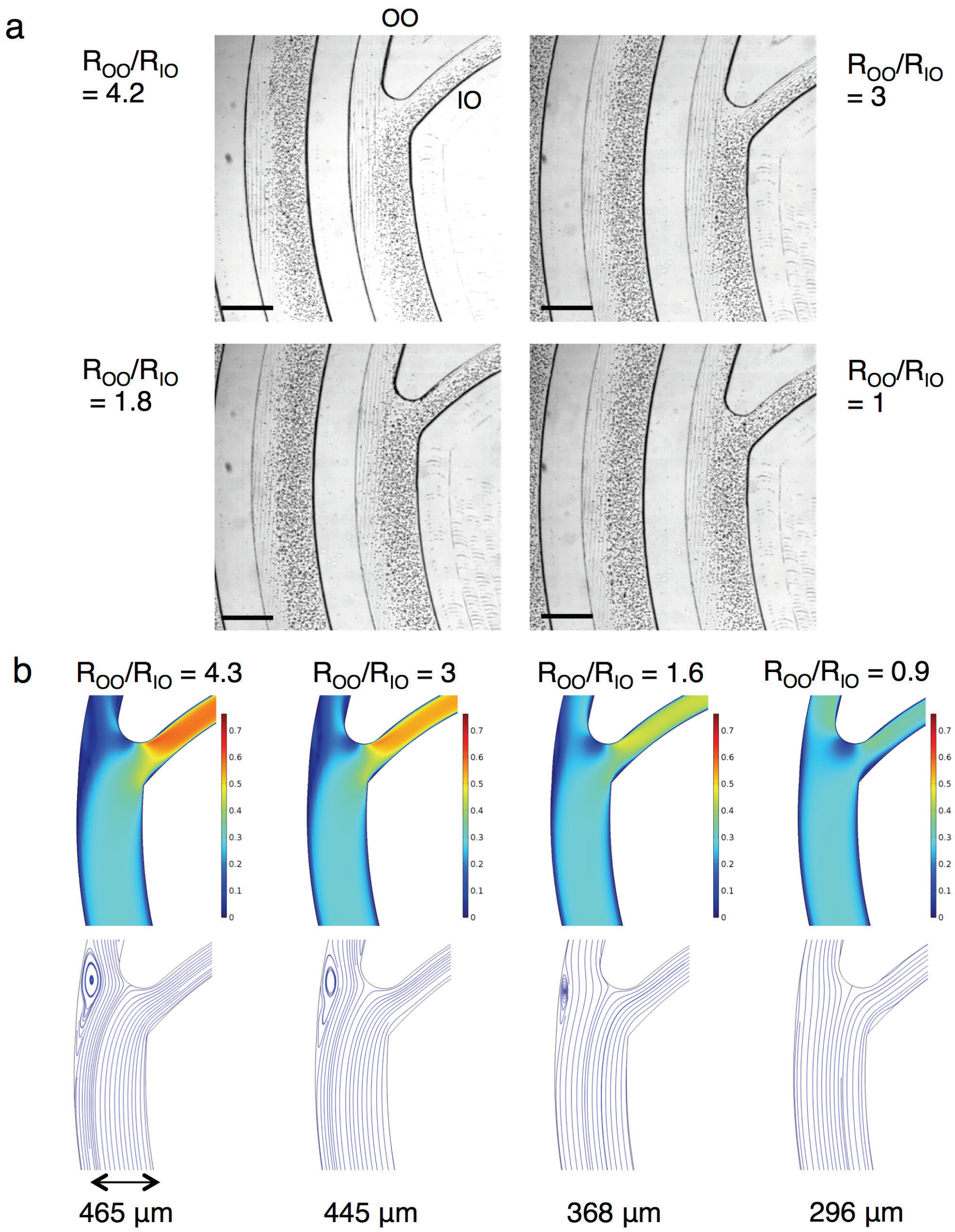

Supplementary Figure S2 The CHO cells at the bifurcation of the microfluidic cell retention device at different fluidic resistance ratios of the outlets **(a)** The CHO cells (11 million cells/mL, 97% viability) near the outlets of the microfluidic cell retention device (R_{OO}, fluidic resistance of the outer outlet; R_{IO}, fluidic resistance of the inner outlet). The input flow rate was 1 mL/min. Higher cell retention was visually confirmed for higher fluidic resistance ratio (R_{OO}/R_{IO}). Scale bars, 500 µm. The distinct clear region in the inner outlet was identified at the first and second highest fluidic resistance ratios (R_{OO}/R_{IO} = 4.2 and R_{OO}/R_{IO} = 3). This region indicates the enlarged streamline boundary at the bifurcation under higher fluidic resistance ratios (R_{OO}/R_{IO}),

because the stream for the inner outlet contained both sorted cells and cell-limited portion of the flow. The stream for the inner outlet at lower fluidic resistance ratios (R_{OO}/R_{IO} = 1.8 and R_{OO}/R_{IO} = 1) contained only the portion of the sorted cells possibly because of the reduced streamline boundary toward the inner wall of the channel. **(b)** The 2D spiral channel designs were constructed to theoretically validate the experimental results using COMSOL simulation. Four spiral channel designs with different fluidic resistance ratios at the outlets were tested using COMSOL software (COMSOL Multiphysics, Burlington, MA, USA). The average input flow velocity was 0.264 m/s. This value corresponds to 1 mL/min flow rate for the spiral channel (600 µm width, 80 µm inner wall depth, 130 µm outer wall depth, and eight loops). The flow velocity of the inner outlet decreased with decreasing the fluidic resistance ratio (R_{OO}/R_{IO}). The 2D streamline plots were obtained to observe changes in the streamline boundary between the inner and outer outlet flows with varying the fluidic resistance ratio at the outlets. The streamline boundary moved toward the inner wall of the channel near the outlets with decreasing the fluidic resistance ratio (R_{OO}/R_{IO}). The width of the inner outlet stream was measured. The channels 1 and 4 measured 465 and 296 µm, respectively. This simulation result showed that the cell retention of the device could be affected by the fluidic resistance ratio at the outlets. Higher resistance ratio (R_{OO}/R_{IO}) resulted in higher cell retention through the inner outlet of the device by increasing the streamline boundary toward the outer wall to induce more focused cells to flow into the inner outlet of the device.

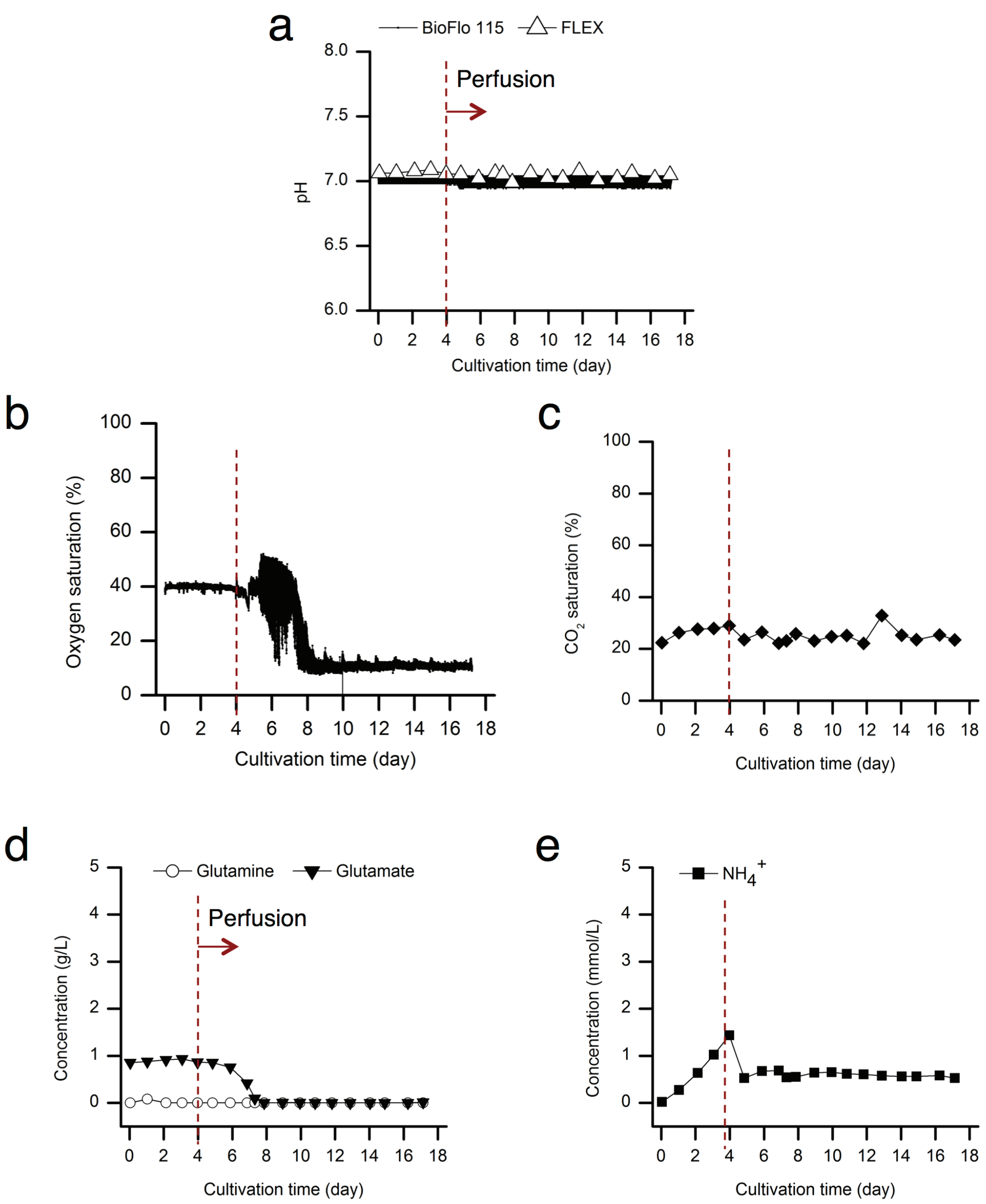

Supplementary Figure S3 Other perfusion culture parameters. **(a)** pH values obtained from two pieces of equipment (BioFlo/CelliGen 115 systems, New Brunswick Scientific, USA; Bioprofile FLEX Analyzer, Nova Biomedical, USA). **(b)** Dissolved oxygen level. **(c)** Dissolved carbon dioxide level. **(d)** Glutamine and glutamate concentrations. **(e)** Ammonium concentration.

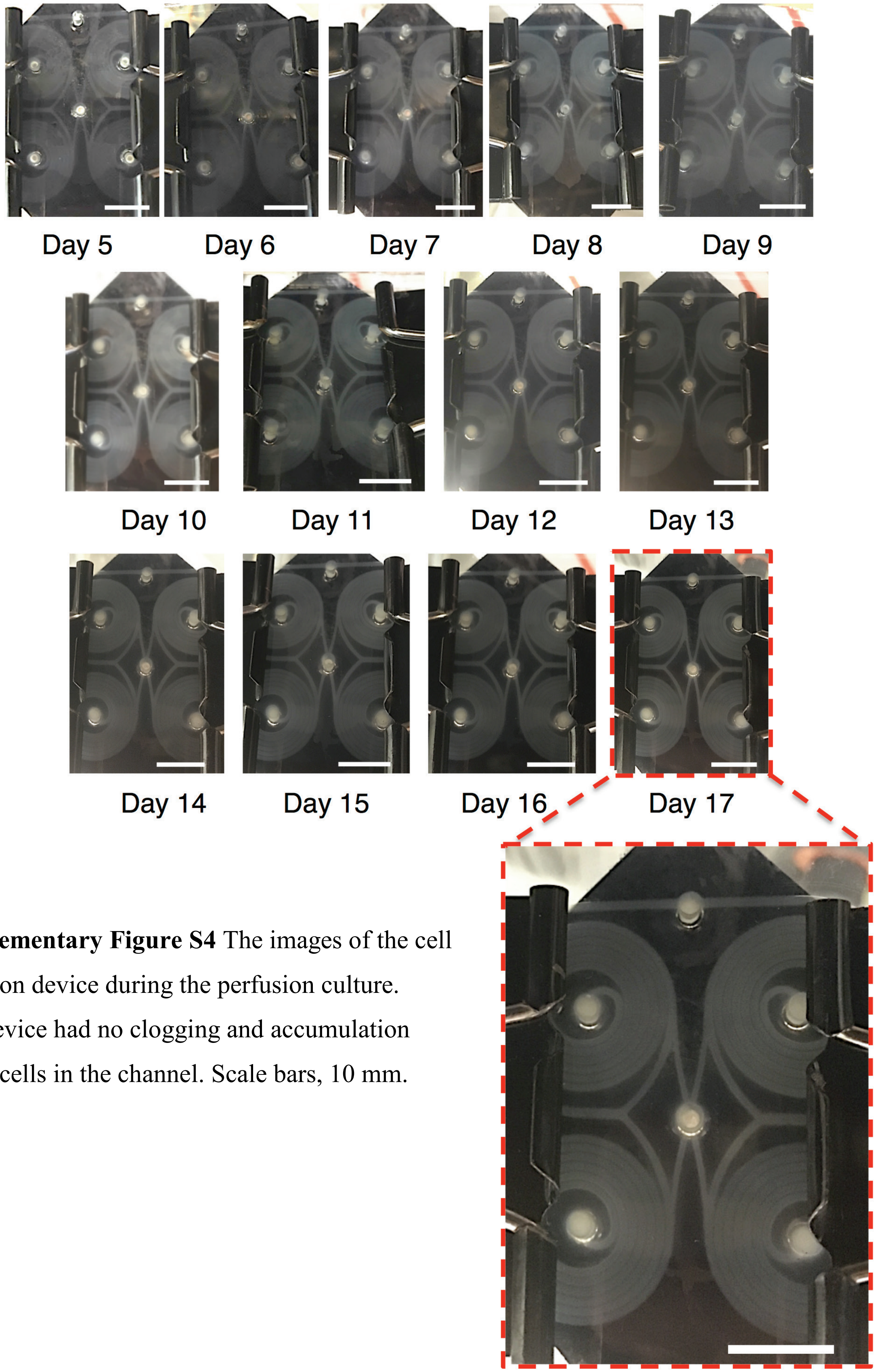

Supplementary Figure S4 The images of the cell retention device during the perfusion culture. The device had no clogging and accumulation of the cells in the channel. Scale bars, 10 mm.

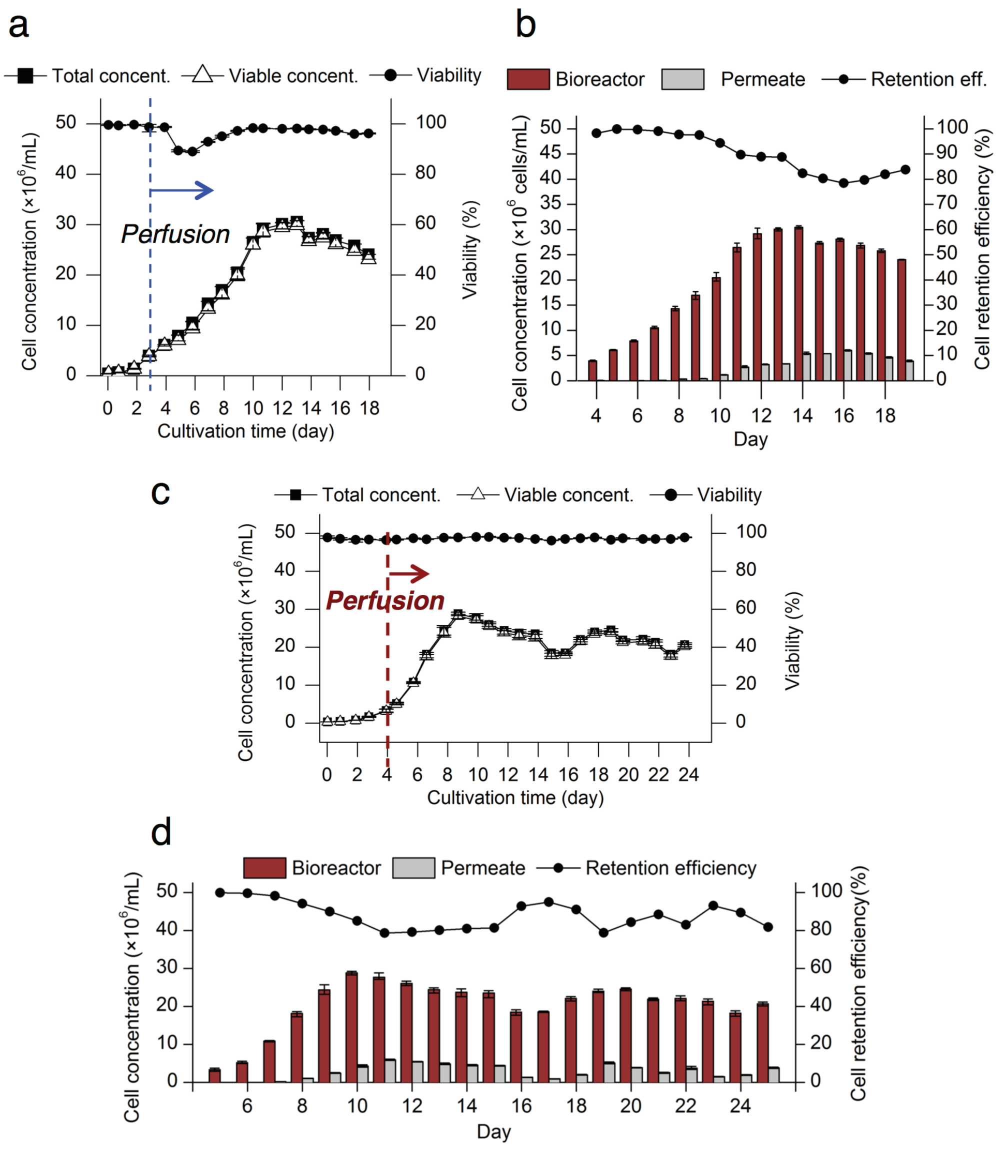

Supplementary Figure S5 The second and third perfusion culture results. Error bars, data range (*n* = 3, technical replicates). **(a)** The decreased viability was observed around Day 5 because of operation failure of the magnetic stirrer. The CHO cells were recovered, and high viability (>97%) was maintained throughout the culture. The maximum cell concentration was approximately 30.5 million cells/mL on Day 13. **(b)** The cell retention efficiency of the device was measured at >80% under the concentration of 20–30 million cells/mL throughout the perfusion culture. **(c)** The peak cell concentration was 28.8 million cells/L, and the average viability during perfusion was 97%. **(d)** The average cell concentrations in the bioreactor and in the permeate after Day 12 were 21.8 and 3.1 million cells/mL, respectively. The average cell retention efficiency after Day 12 was 86%.

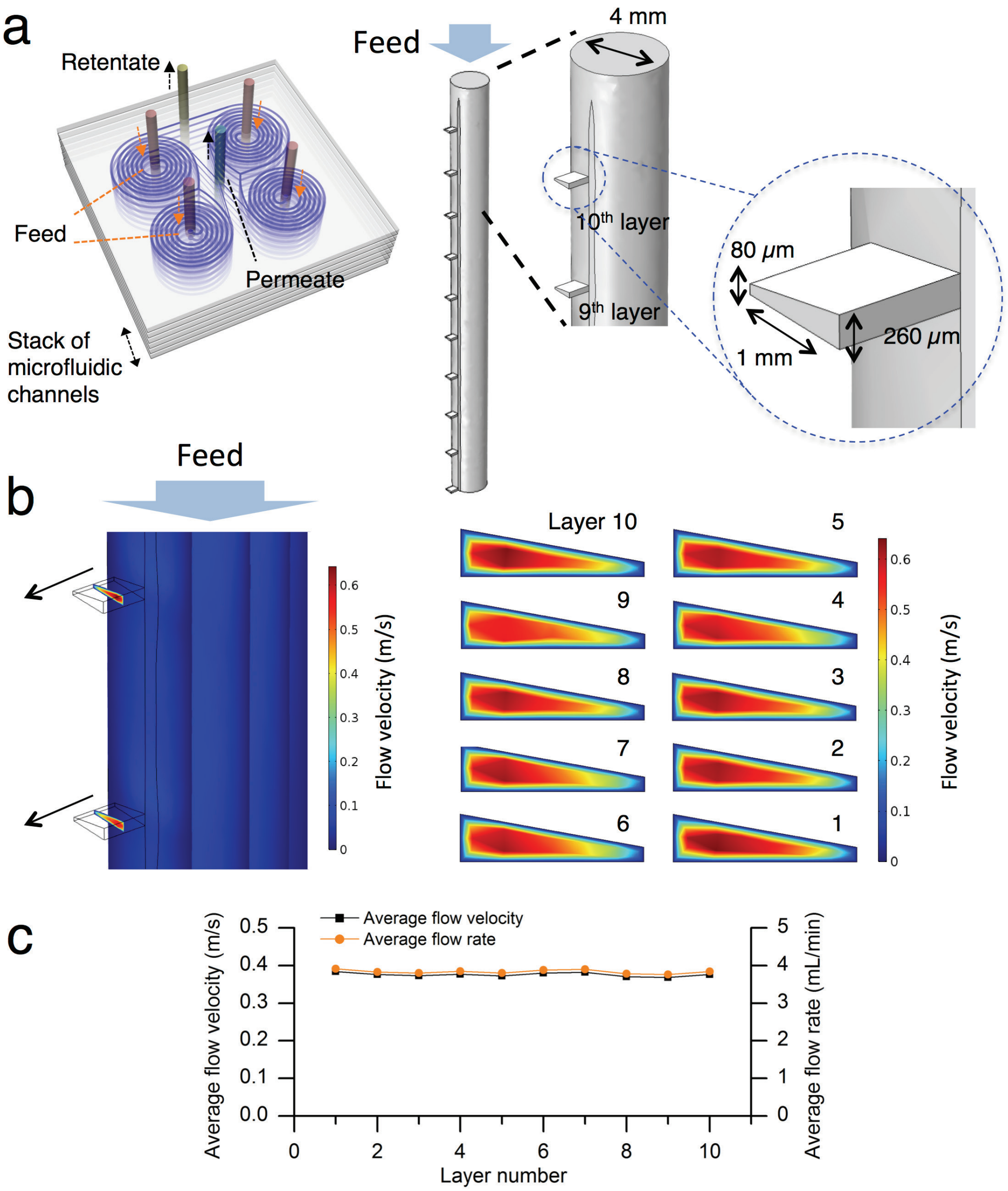

Supplementary Figure S6 Flow distribution in the stacked microfluidic cell retention device. **(a)** One of the feed streams of 10-layer stacked device containing four spiral channels in one layer was simulated using COMSOL software (COMSOL Multiphysics, Burlington, MA, USA). The fluidic paths of common feed stream of 4mm in diameter and inlet streams of 10 spiral channels were modeled. **(b)** The feed velocity of 51.6 mm/s corresponding to 38.9 mL/min flow rate (56 L/day) was simulated, and flow velocity magnitude of each input layer was plotted. **(c)** The surface average of flow velocity magnitude of each input layer was evaluated and converted to the average flow rate. The average flow rate of each layer had the average of 3.83 mL/min and standard deviation of 0.05 mL/min.

Supplementary Table S2 Summary of the batch and perfusion cultures.

Parameter	Batch #1	Batch #2	Perfusion #1
Cultivation time (h)	264	264	390
Perfusion time (h)	N/A	N/A	294
Working volume (mL)	250	250	350
Spent medium (mL)	250	250	8510
Maximum viable cell concentration ($\times10^6$ cells/mL)	4.8	5.7	22.7
Maximum total cell concentration ($\times10^6$ cells/mL)	5.5	6.1	23.0
Average specific growth rate during exponential growth phase (h^{-1}) [†]	0.03	0.03	0.03
Total amount of product harvested (mg)	8.2	8.9	262.6
IgG1 titer (mg·spent medium L^{-1})	32.8	35.6	30.9
Integral cell area ($\times10^6$ cells·mL^{-1}·day) [§]	33.9	37.5	228.4
Specific productivity (pg·$cell^{-1}$·day^{-1})[*]	1.0	0.9	3.3
Bioreactor productivity (mg·working volume L^{-1}·day^{-1})	3.0	3.2	46.9

[†]The specific cell growth rate during exponential growth phase was calculated as follows: $\mu = \frac{\ln(X_2)-\ln(X_1)}{t_2-t_1}$, where μ, X, and t represent the growth rate, the viable cell concentration, the time at which the sample was taken, respectively.

[§]Area under the curve for cell growth that accounts for the titer

[*]Titer/(Integral cell area) for the batch cultures, (Titer × spent medium volume)/(Integral cell area × working volume) for the perfusion culture

Supplementary Table S3 Comparison with other cell retention technologies commercially available for perfusion cultures of suspended mammalian cells (CHO cells)

	**Tangential Flow Filtration (TFF)**	**Alternating Tangential Flow System (ATF)**	**Inertial sorting**	**Centrifugation**	**Acoustic wave**	**Inclined sedimentation**
Company / Institution	GE Healthcare, Spectrum Labs	Repligen	MIT	Pneumatic Scale Angelus[a], Sartorius[b]	Applikon Biotechnology[a], APIcells[b]	Roche Diagnostics GmbH
Maximum cell density ($\times10^6$ cells/mL)	214[1]	132[1]	44[*]	35[a, 2]<br>15[b, 3]	20[a, 4]	16[5]
Cell retention efficiency (%)	100	100	84[*] to 99	Rapid decrease observed at >40×10^6 cells/mL[2]	Rapid decrease observed at >20×10^6 cells/mL[a, 4]	85 to 99[5]
Scalability (L/day)	2 to 2000[6]	<1 to >2000[6]	<1 to >500	Up to 3000[a, 6]<br>100 to 6000[b, 6]	<1 to 1000[a, †]<br>1 to 20[b, §]	Up to 3000[5]
Product recovery	Low	Medium	High	High	High	High
Dead cell removal	No	No	Yes	Yes	Yes	Yes
Capital and operation cost	High	High	Low	High	Medium	Low

[*] Obtained from the *in vitro* device characterization experiment (**Fig. 2d**)

[†] Retrieved November 29, 2016 from http://www.applikonbio.com/en/products/biosep-perfusion

[§] Retrieved November 29, 2016 from http://www.apicells.com/news/105-the-cytoperf-technology-at-the-esact

REFERENCES

1. Clincke, M. F. *et al.* Very high density of CHO cells in perfusion by ATF or TFF in WAVE bioreactor™: Part I: Effect of the cell density on the process. *Biotechnol. Prog.* **29,** 754–767 (2013).
2. Kim, S. C. *et al.* Effect of transmembrane pressure on Factor VIII yield in ATF perfusion culture for the production of recombinant human Factor VIII co-expressed with von Willebrand factor. *Cytotechnology* **68,** 1687–1696 (2016).
3. Mehta, S. in *Continuous Processing in Pharmaceutical Manufacturing* (ed. Subramanian, G.) 385–400 (2014). doi:10.1002/9783527673681.ch15
4. Duvar, S., Hecht, V., Finger, J., Gullans, M. & Ziehr, H. Developing an upstream process for a monoclonal antibody including medium optimization. *BMC Proc.* **7,** P34 (2013).
5. Pohlscheidt, M. *et al.* Optimizing capacity utilization by large scale 3000 L perfusion in seed train bioreactors. *Biotechnol. Prog.* **29,** 222–229 (2013).
6. Whitford, W. G. in *Continuous Processing in Pharmaceutical Manufacturing* (ed. Subramanian, G.) 183–226 (2014). doi:10.1002/9783527673681.ch09
